# CB_1_ cannabinoid receptor enrichment in the ependymal region of the adult human spinal cord

**DOI:** 10.1038/srep17745

**Published:** 2015-12-04

**Authors:** Beatriz Paniagua-Torija, Angel Arevalo-Martin, Isidro Ferrer, Eduardo Molina-Holgado, Daniel Garcia-Ovejero

**Affiliations:** 1 Laboratory of Neuroinflammation, Hospital Nacional de Paraplejicos (SESCAM), Toledo, Spain; 2 Institut de Neuropatologia, Servei d’Anatomia Patològica, IDIBELL-Hospital Universitari de Bellvitge, Universitat de Barcelona, L’Hospitalet de Llobregat, Spain

## Abstract

Cannabinoids are involved in the regulation of neural stem cell biology and their receptors are expressed in the neurogenic niches of adult rodents. In the spinal cord of rats and mice, neural stem cells can be found in the ependymal region, surrounding the central canal, but there is evidence that this region is largely different in adult humans: lacks a patent canal and presents perivascular pseudorosettes, typically found in low grade ependymomas. Using Laser Capture Microdissection, Taqman gene expression assays and immunohistochemistry, we have studied the expression of endocannabinoid system components (receptors and enzymes) at the human spinal cord ependymal region. We observe that ependymal region is enriched in CB_1_ cannabinoid receptor, due to high CB_1_ expression in GFAP+ astrocytic domains. However, in human spinal cord levels that retain central canal patency we found ependymal cells with high CB_1_ expression, equivalent to the CB_1_^HIGH^ cell subpopulation described in rodents. Our results support the existence of ependymal CB_1_^HIGH^ cells across species, and may encourage further studies on this subpopulation, although only in cases when central canal is patent. In the adult human ependyma, which usually shows central canal absence, CB_1_ may play a different role by modulating astrocyte functions.

The Endocannabinoid System (ECBS) is formed by lipid ligands (endocannabinoids), the enzymatic machinery for their synthesis and degradation and their specific G-protein coupled CB_1_ and CB_2_ receptors. The most important endocannabinoids are 2-arachydonoylglycerol (2-AG) and anandamide (AEA)^1^. These compounds are involved in the control of neural stem cell biology^2^, and many of their effects are mediated by the cannabinoid receptor CB_1_. CB_1_ receptor is expressed in all neurogenic niches in rodents, including the ependymal region of the spinal cord (reviewed in^3^). In this region, that holds neural stem cell potential^4^,^5^, a subpopulation of cells expresses high levels of CB_1_ receptor (CB_1_^HIGH^ cells), and proliferate after lesion or during postnatal development in rats^6^. However, the ependymal region of the adult human spinal cord is strikingly different from that of rodents and other primates: although it contains ependymal cells, lacks a patent central canal and shows perivascular pseudorosettes^7^,^8^,^9^. This means that observations made in rodents should be validated in human tissue to understand the composition and the regenerative potential of this niche. Here we have explored the presence of the ECBS and searched for an equivalent of rat and mice CB_1_^HIGH^ cells in adult human spinal cord.

## Results and Discussion

We found that human ependymal region consistently expresses CB_1_ cannabinoid receptor (CNR1 gene; Table 1). CB_1_ receptor could be the target of locally produced 2-AG, since we also found expression (although non enrichment) of enzymes related with 2-AG synthesis and degradation: diacylglycerol lipase α (DAGLA), diacylglycerol lipase β (DAGLB), monoacylglycerol lipase (MGLL) and abhydrolase domain-containing proteins – 6 (ABHD6) and –12 (ABHD12). On the contrary, we could not find consistent expression of enzymes related with direct anandamide synthesis or degradation (NAPE-phospholipase D and fatty acid amide hydrolase, respectively). However, it should be noted that alternative enzymatic routes have been described for AEA, involving glycerophosphodiester phosphodiesterase and N-acylethalnolamine-hydrolyzing acid amidase that have been not tested here^2^. We also did not find expression of CB_2_ cannabinoid receptor or the related GPR55 receptor. In previous works, we observed expression (but not enrichment) of PPAR-α, another cannabinoid-related receptor^1^, in human ependymal region^9^.

**Figure 1:**
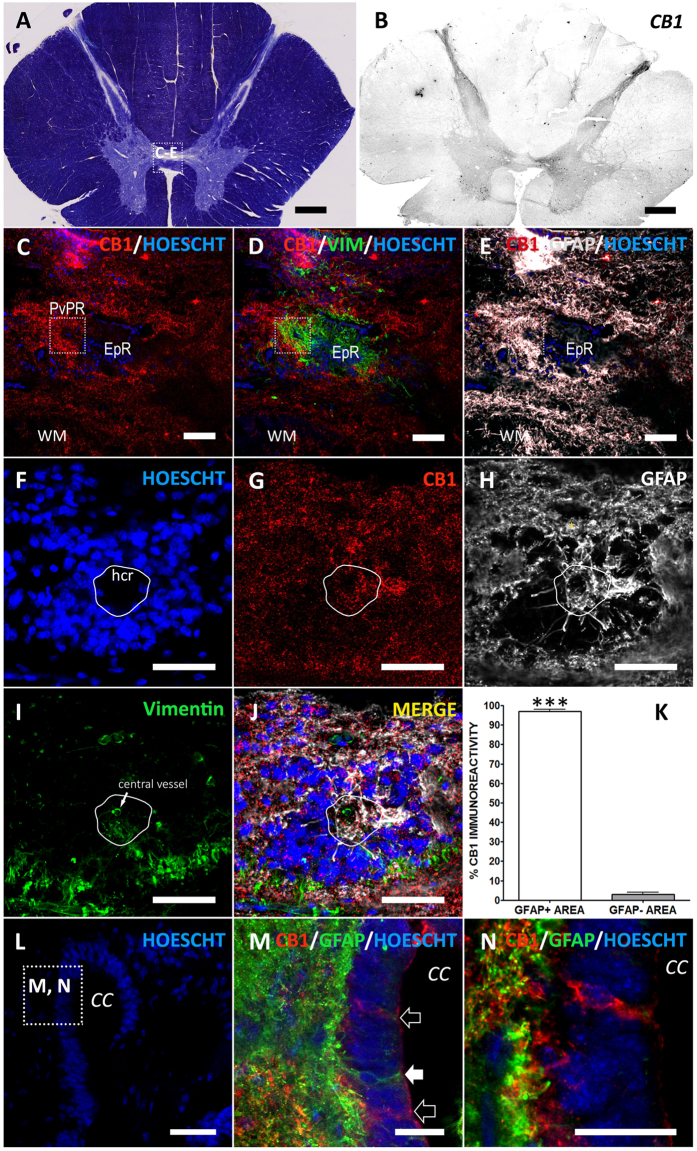
CB_1_ cannabinoid receptor in adult human spinal cord. (**A**) Myelin staining of a representative thoracic spinal cord section. Square depicts the area shown in (**C**–**E**). (**B**) In low magnification a strong CB_1_ immunoreactivity can be found in dorsal horn, lamina X and ventral gray matter. (**C–E**) Higher magnification of central gray shows CB_1_ expression in GFAP+ areas surrounding the Vimentin^+^ cells at the ependymary region (EpR). Square highlights the location of a perivascular pseudorosette (PvPR). (**F–J**) Strong CB_1_ immunoreactivity is found at the GFAP^+^ domains around and inside perivascular pseudorosettes, including the GFAP ribbon at the hypocellular region of the pseudorosette (hcr, outlined in white). In PvPRs cells are arranged around a central vessel ((**I**) arrow). (**K**) Quantification supports qualitative observations: CB1 immunoreactivity is significantly accumulated in GFAP+ areas. (**L**) Detail of the dorsal aspect of an ependymal region with a patent central canal. Square depicts location of images M and N. (**M**) CB_1_^HIGH^ ependymal cells (empty arrows) can be found intermingled with ependymocytes lining the central canal. GFAP^+^ cells contacting central canal lumen (arrowhead) are CB_1_^−^. (**N**) Detail of M, showing a CB_1_^HIGH^ cell with a dim staining of GFAP in the apical region. ***T student, p < *0.001; CC, Central Canal; EpR, ependymal region; hcr, hypocellular region; PvPR, perivascular pseudorosette; WM, white matter. Scale bars: A,B* = *1* *mm; C-E: 100* *μm; F-L* = *50* *μm; M,N* = *25* *μm.*

**Figure 2:**
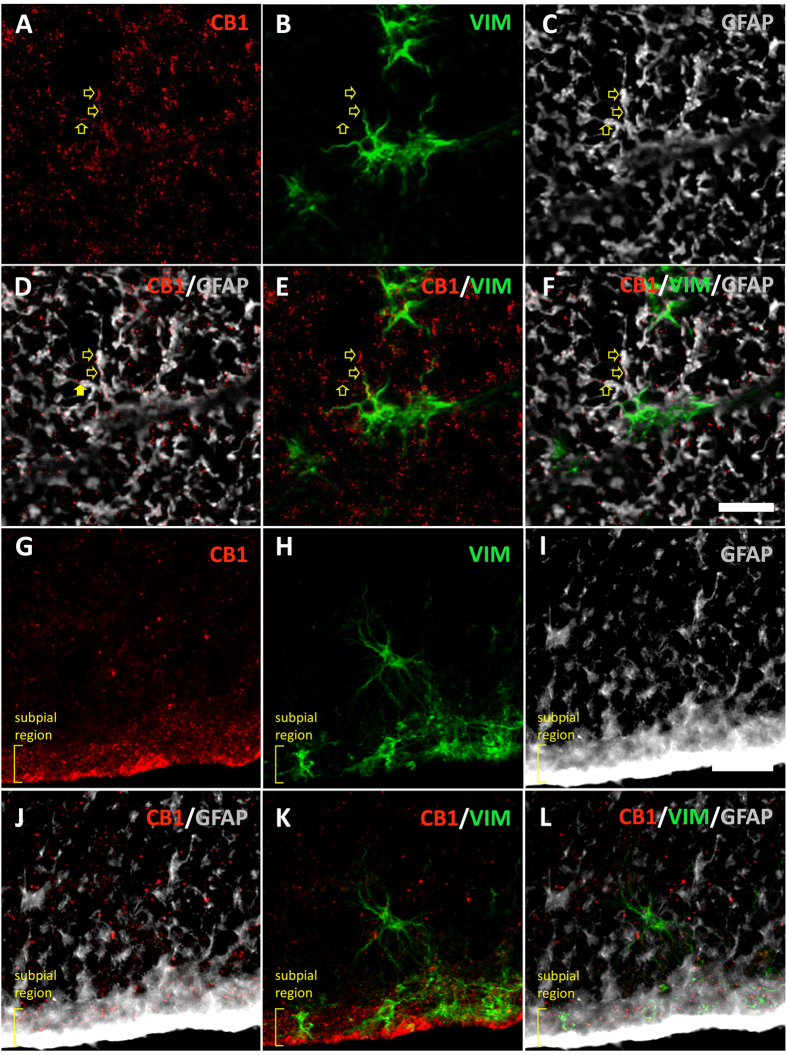
CB_1_ immunoreactivity can be found in astrocytes of other regions in the spinal cord. (**A–F**) CB_1_ is expressed in the processes of GFAP^+^ and Vim^+^ astrocytes (arrows) at the dorsolateral white matter. (**G–I**) A strong CB_1_ expression can be found in astrocytic processes at the subpial region. *Scale bars: 25* *μm*.

When compared with ventral horn, only CNR1 (CB_1_ receptor) was significantly enriched at the ependymal region (Table 1). Accordingly, we found a strong CB_1_ immunoreactivity in central gray matter by immunohistochemistry (Fig. 1B–J). But CB_1_ enrichment in adult humans ependyma is not equivalent to that found in rodents: In humans, CB_1_ is expressed by astrocytes, forming part of the gliosis that accompanies central canal closure (Fig. 1C–E) and in the GFAP^+^ hypocellular ribbon of perivascular pseudorosettes (Fig. 1F–K)^9^,^10^. CB_1_ receptor is also expressed in astrocytes from other spinal cord areas (Fig. 2), and its intensity is apparently related to high GFAP expression. Accordingly, a strong CB_1_ expression has been reported in reactive astrocytes of human pathologies like spinocerebellar ataxia^11^ or temporal lobe epilepsia^12^. The role of astrocytic CB_1_ could be multiple: protection^13^, metabolism increase^14^, control of inflammation^15^,^16^,^17^, inhibition of glutamate transporters^18^ or release of neurotransmitters such as glutamate^19^, ATP and D-serine^20^.

Interestingly, we obtained some sections from adult individuals in which parts of the central canal were patent. In those sections, we found ependymal cells with high expression of CB_1_ receptors lining the canal (Fig. 1L–N), resembling those CB_1_^HIGH^ cells described for rats and mice^6^. These cells were mostly GFAP-, except for a very dim expression at the apical pole (Fig. 1N), in contrast with strongly GFAP^+^ cells embeded in the ependymal layer (Fig. 1M).

Our results support the existence of ependymal CB_1_^HIGH^ cells across species, and may encourage further studies on this subpopulation, although only in cases when there is central canal patency, i.e. childhood and upper cervical levels^8^,^9^. But in the majority of adult ependyma, CB_1_ is enriched in astrocyte domains, and cannabinoids may play a different role, that still might be relevant, in terms of homeostasis maintenance and response to injury.

## Methods

Human tissue was obtained from the HUFA BioBank (Alcorcon, Spain) and the HUB-ICO-IDIBELL BioBank (Hospitalet de Llobregat, Spain). Samples were obtained from donor individuals deceased without clinical or histopathological involvement of the spinal cord (Table 2). Donation always included a written informed consent from donors while alive or from their families after death. Data from donors and handling of samples were carried out after approval by the Clinical Research Ethical Committee (CEIC) in Toledo (Spain), in accordance with the Spanish law and International Guidelines (LOPD 15/1999; RD 1720/2007; Helsinki declaration, 2008).

### Gene expression in human ependymal region

All procedures were performed according to our published protocol^9^. Briefly, fresh frozen spinal cord blocks were cut in 25 μm thick sections and the ependymal region microdissected with a Laser Dissection Microscope. RNA extraction, amplification and reverse transcription were performed as previously described^9^. We also collected microdissected portions of ventral horn, which we used as a non-neurogenic, non-ependymal reference for gene expression.

Gene expression was studied with Taqman PCR Assays (Life Technologies, Madrid, Spain) either incorporated in Taqman Low Density Arrays (*DAGLA, #Hs00391374_m1; DAGLB, #Hs00373700_m1; MGLL, #Hs00200752_m1; NAPEPLD, #Hs00419593_m1*) or in individual assays (*ABHD6, #Hs00977889_m1; ABHD12, #Hs01018047_m1; CNR1, #Hs01038522_s1; CNR2, #Hs00361490_m1; FAAH, #Hs01038660_m1; GPR55, #Hs00271662_s1*). We used 18S gene as an endogenous control (*18S, #Hs03003631_g1*). For assays incorporated on TLDAs, we added 1.25 ng cDNA/well. For assays performed individually, we added 1.5 ng cDNA/well. Assays were run on an Applied Biosystems® 7900HT Fast Real-Time PCR System. Data were analysed as described^9^ using automatic detection of Ct, normalized with the endogenous gene (ΔCt vs 18S). Only genes expressed in at least three out of four samples were considered as consistently expressed and included in statistics. Enrichment was defined as higher and statistically significant expression in ependymal region vs ventral horn (Student’s t-test with ΔCts, p < 0.05). To obtain folds of enrichment, we used Relative quantity formula, RQ = 2^−ΔΔCt.

### Immunohistochemistry

To improve signal to noise ratio and avoid autofluorescence, we amplified CB_1_ immunoreactivity using Tyramide Signal Amplification System (TSA Plus Cyanine 3 System #NEL744001KT, Perkin Elmer, USA). Free floating vibratome sections (40 μm) were rinsed on 0.1 M phosphate-buffered saline containing 0.5% bovine serum albumin +0.3% Triton X-100. Endogenous peroxidase inhibition and antigen demasking were performed as described^9^. Sections were then blocked with TSA Blocking Solution (45′) and incubated for 2 days with primary antibodies diluted in rinse solution +10% Normal Donkey Serum: guinea pig anti-CB_1_ (1:2000, #CB1-GP-Af530-1, FSI, Japan), rabbit anti-GFAP (1:2000, #Z0334, DAKO, Spain) and mouse anti-Vimentin (1:300, #M0725, DAKO, Spain). Immunoreactivity was visualized by incubating sections with Alexa 488-, Alexa 555- and Alexa 633- secondary antibodies (1:1000, Invitrogen, Spain) or horseradish peroxidase donkey anti-guinea pig antibody (1:300, Jackson Immunoresearch, UK) followed by Tyramide-Cy3 diluted in TSA Amplification Buffer (1:50). Samples were analyzed with a LEICA SP5 confocal microscope. We ruled out the interference of nonspecific staining by omitting primary antibodies. We set the confocal parameters at a point where no signal was observed in these primary antibody controls and those settings were used for all the image acquisitions (Supplementary Figure 1A–F). Furthermore, as discussed in several reports, there is a variety of antibodies against CB_1_ receptor, and some of them may show non-specific staining^21^,^22^,^23^. The specificity of CB_1_ antibody used for this report has been extensively validated by other laboratories and ourselves in previous works^6^,^24^,^25^. We show here an additional validation in the Supplementary Figure 1 by using immunohistochemistry and TSA amplification on wild type (C57BL/6N) and CB_1_ knockout mice tissue (kindly donated by Dr. Galve-Roperh^26^). Using restrictive confocal parameters (as we did for humans), we got rid out of autofluorescence, background staining and most of the non-specific staining observed in the knockout mice that, in these conditions, is limited to a dim intracellular neuronal staining, largely different from that observed in the wild type mice (Supplementary Figure 1L-Q). All post-capture image modifications were identically performed for controls, including cropping, noise reduction and minor adjustments to optimize contrast and brightness.

To quantitatively support CB_1_ enrichment in the astrocytic area, we calculated the fraction of CB_1_ found in GFAP^+^ vs GFAP^−^ areas on confocal planes (image size 190 μm × 190 μm) using Fiji (http://pacific.mpi-cbg.de). For this, we outlined GFAP borders using manual Threshold with Otsu Filter and used this ROI on the CB_1_ image corresponding to the same confocal plane. We measured CB_1_^+^ Area inside and outside the selection (GFAP^+^ and GFAP^−^ areas, respectively) and expressed them as % of total CB_1_ staining (Fig. 1K). We used Student T-test for statistical comparisons.

## Additional Information

**How to cite this article**: Paniagua-Torija, B. *et al.* CB_1_ cannabinoid receptor enrichment in the ependymal region of the adult human spinal cord. *Sci. Rep.* **5**, 17745; doi: 10.1038/srep17745 (2015).

## Supplementary Material

Supplementary Figure 1

## Figures and Tables

**Table 1:** Relative expression of endocannabinoid system related genes in the adult human ependymal region compared with ventral horn.

*GENE*	*Assay*	Δ*Ct Ependymal Region vs18S* (*average*)	ΔC*t Ventral Horn vs18S* (*average*)	*RQ vs Ventral Horn*	*p*
**CNR1**	*Hs01038522_s1*	*8,379754298*	*13,57232086*	**57,27**	0,012*
CNR2	*Hs00361490_m1*	–	–	*ND*^#^	–
GPR55	*Hs00271662_s1*	*17,13233*	*18,721182*	*NCD*^#^	–
DAGLA	*Hs00391374_ml*	*11,7639845*	*13,04953063*	2,34	0,8
DAGLB	*Hs00373700_ml*	*11,1895161*	*8,199532*	14,6	0,51
MGLL	*Hs00200752_ml*	*10,5709992*	*9,519471*	0,25	0,44
ABHD6	*Hs00977889_m1*	*12,6129394*	*14,365685*	1,48	0,75
ABHD12	*Hs01018047_m1*	*12,6756672*	*12,6676875*	4,7	0,095
NAPEPLD	*Hs00419593_m1*	*13,0519701*	*6,908164*	*NCD*#	–
FAAH	*Hs01038660_m1*	*20,2593*	*19,962125*	*NCD*#	–

^*^Significantly enriched in Ependymal region vs Ventral Horn (Student T-test).

^#^ND: Non detected in the Ependymal region of any individual; NCD: Non consistently detected (detected in less than 3 of the 4 individuals).

**Table 2:** Postmortem Spinal Cord tissue samples used for immunohistochemistry (IHC) and/or Laser Capture Microdissection (LCMD).

Autopsy number	Cause of Death	Gender	Age	Coded as	Postmortem delay	Used for
BC01015	Unknown. No significant neuropathological alterations in the spinal cord	Male	60	Control	Unknown	IHC
BC01684	Acute Hypoxia-ischemia	Male	27	Control	Unknown	IHC
A07/00044	Cardiopulmonary arrest	Male	39	Control	3 h 30 min	IHC
A07/00067	Refractary septic shock and cardiac arrest. Ischemic cardiopathy	Male	47	Control	4 h 55 min	IHC
A10/00017	Hepatic metastasis. Probable pancreatic neoplasia	Male	52	Control	03 h	IHC
A07/00041	Multiorganic failure. Gastric tumour	Male	43	Control	5 h 55 min	IHC, LCMD
A07/00084	Refractary septic shock	Male	46	Control	15 h	IHC, LCMD
A10/00026	Multiorganic failure. Severe broncopathy	Male	61	Control	3 h 55 min	LCMD
A05/00134	Carcinoma and metastasis. With brain but not spinal cord metastasis.	Female	32	Control	11 h 45 m	LCMD
A11/00052	Endocarditis. No neuropathological features	Male	76	Control	06 h 30 m	LCMD
A12/00046	Cardiac arrest. No neuropathological features	Female	75	Control	06 h10 m	LCMD
