# Supplementary material for: CB1 cannabinoid receptor enrichment in the ependymal region of the adult human spinal cord
Source: Sci Rep. 2015 Dec 4;5:17745. doi: 10.1038/srep17745 (PMC4669459; doi:10.1038/srep17745)
Supplement: Supplementary Figure 1 [file srep17745-s1.pdf]

SUPPLEMENTARY MATERIAL

CB_1 cannabinoid receptor enrichment in the ependymal region of the adult human spinal cord

Beatriz Paniagua-Torija[1], Angel Arevalo-Martin[1], Isidro Ferrer[3], Eduardo Molina-Holgado[1]*, Daniel Garcia-Ovejero[1]*,

[1]Laboratory of Neuroinflammation, Hospital Nacional de Paraplejicos (SESCAM), Toledo, Spain

[3]Institut de Neuropatologia, Servei d'Anatomia Patològica, IDIBELL-Hospital Universitari de Bellvitge, Universitat de Barcelona, L'Hospitalet de Llobregat, Spain

Corresponding authors: Eduardo Molina-Holgado, Ph.D., Daniel Garcia-Ovejero, Ph.D., Laboratory of Neuroinflammation, Hospital Nacional de Paraplejicos, Finca La Peraleda s/n, 45071-Toledo, Spain. Ph: +34925247754, Fax: +34925247745; eduardom@sescam.jccm.es, dgarciao@sescam.jccm.es;

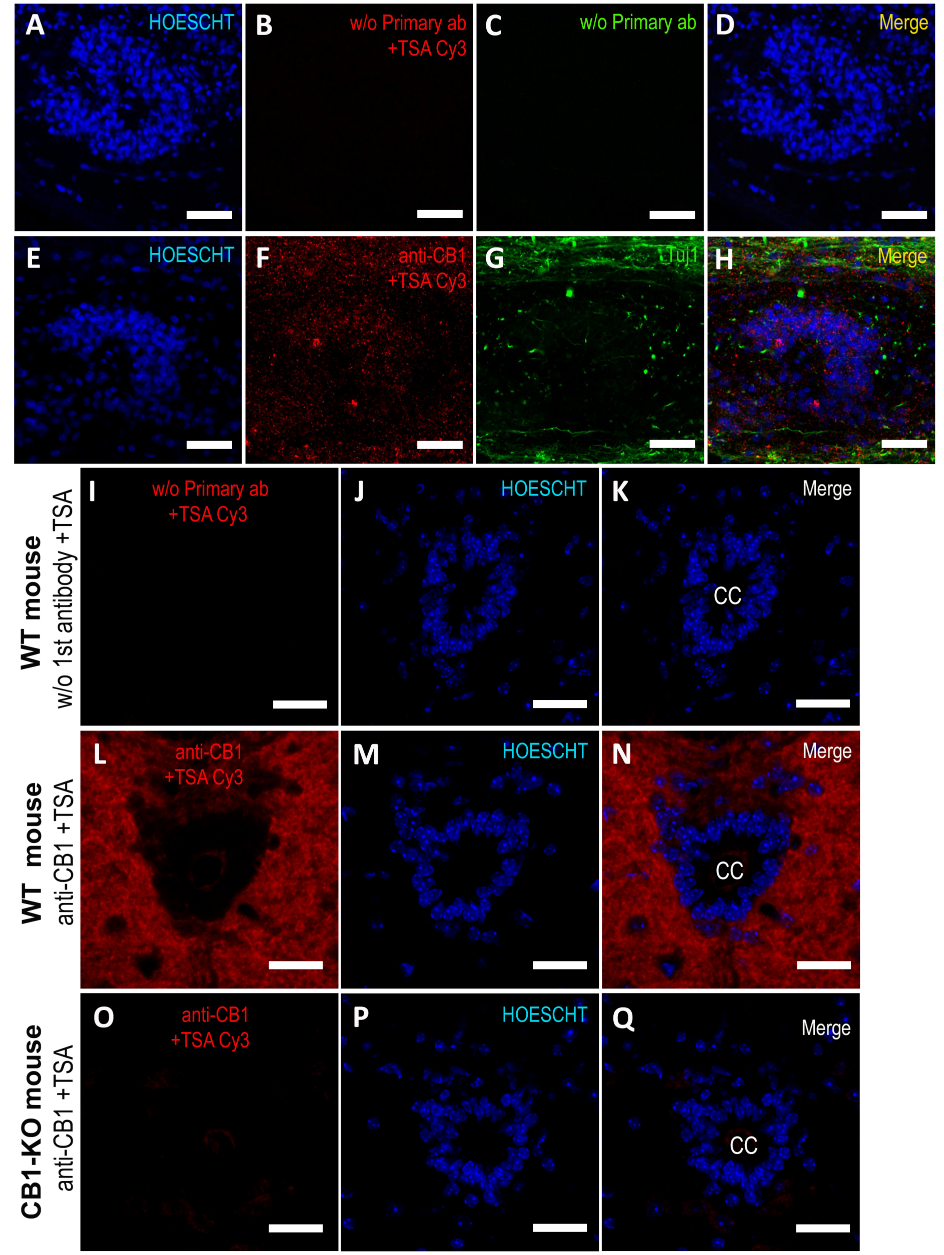

Suplementary Figure 1. Representative images of controls for background signal and CB1 antibody specificity. **(A-H)** Absence of primary antibodies completely abolishes CB1 immunoreactivity in human tisue after amplification with Cy3-Tyramide amplification System (TSA-Cy3) . BetaIII tubulin + axons are shown in green. **(I-K)** Background staining is also absent in C57/BL6 mice after ommitting CB1 antibody using the same staining protocol and restrictive microscope settings. **(L-N)** When using TSA, a very intense CB1 immunoreactivity is observed in C57/BL6 mice that is in acordance to the pattern previously described (Garcia-Ovejero et al., 2013). Images show CB1 staining in ependymal region. **(O-Q)** When using the same microscope settings, only a dim staining can be found in CB1 knockout mice, supporting the specificity of this CB1 antibody under our the conditions used here. *CC, central canal. Magnification bars: A-H = 50 µm; I-Q = 25 µm.*
